# Primary prevention implantable cardioverter defibrillator in cardiac resynchronization therapy recipients with advanced chronic kidney disease

**DOI:** 10.3389/fcvm.2023.1237118

**Published:** 2023-08-23

**Authors:** Ido Goldenberg, Valentina Kutyifa, Wojciech Zareba, David Tsu-Chau Huang, Spencer Z. Rosero, Arwa Younis, Claudio Schuger, Anna Gao, Scott McNitt, Bronislava Polonsky, Jonathan S. Steinberg, Ilan Goldenberg, Mehmet K. Aktas

**Affiliations:** ^1^ University of Rochester Medical Center, Rochester, NY, United States; ^2^ Rochester General Hospital, Rochester, NY, United States

**Keywords:** cardiac resynchronization therapy, implantable cardioverter defibrillator, ventricular arrhythmia, chronic kidney disease, sudden cardiac death

## Abstract

**Introduction:**

The implantable cardioverter defibrillator (ICD) is effective for the prevention of sudden cardiac death (SCD) in patients with heart failure and a reduced ejection fraction (HFrEF). The benefit of the ICD in patients with advanced CKD, remains elusive. Moreover, the benefit of the ICD in patients with advanced chronic kidney disease (CKD) and HFrEF who are cardiac resynchronization therapy (CRT) recipients may be attenuated.

**Hypothesis:**

We hypothesized that patients with CKD who are CRT recipients may derive less benefit from the ICD due to the competing risk of dying prior to experiencing an arrhythmia.

**Methods:**

The study population included 1,015 patients receiving CRT with defibrillator (CRT-D) device for primary prevention of SCD who were enrolled in either (Multicenter Automated Defibrillator Implantation Trial) MADIT-CRT trial or the Ranolazine in High-Risk Patients with Implanted Cardioverter Defibrillator (RAID) trial. The cohort was divided into two groups based on the stage of CKD: those with Stage 1 to 3a KD, labeled as (S1-S3a)KD. The second group included patients with Stage 3b to stage 5 kidney disease, labeled as (S3b-S5)KD. The primary endpoint was any ventricular tachycardia (VT) or ventricular fibrillation (VF) (Any VT/VF).

**Results:**

The cumulative incidence of Any VT/VF was 23.5% in patients with (S1-S3a)KD and 12.6% in those with (S3b-S5)KD (*p* < 0.001) The incidence of Death without Any VT/VF was 6.6% in patients with (S1-S3a)KD and 21.6% in patients with (S3b-S5)KD (*p* < 0.001). A Fine and Gray multivariate competing risk regression model showed that Patients with (S3b-S5)KD had a 43% less risk of experiencing Any VT/VF when compared to those with (S1-S3a)KD (HR = 0.56, 95% CI [0.33–0.94] *p* = 0.03. After two years of follow up, there was almost a 5-fold increased risk of Death without Any VT/VF among patients with (S3b-S5)KD when compared to those with (S1-S3a)KD [HR = 4.63, 95% CI (2.46–8.72), *p* for interaction with time = 0.012].

**Conclusion:**

Due to their lower incidence of arrhythmias and higher risk of dying prior to experiencing an arrhythmia, the benefit of the ICD may be attenuated in CRT recipients with advanced CKD. Future prospective trials should evaluate whether CRT without a defibrillator may be more appropriate for these patients.

## Introduction

The implantable cardioverter-defibrillator has been shown to be effective for the prevention of sudden cardiac death (SCD) in patients with heart failure and a reduced ejection fraction (HFrEF) across many randomized controlled trials (1, 2). However, the benefit of an ICD is not uniform due to presence of comorbidities that effect survival in patients with HFrEF. More recently, we developed the Multicenter Automated Defibrillator Implantation Trial (MADIT)-ICD score, which has since been validated, as a useful tool in identifying patients who are mostly likely to benefit from prophylactic ICD implantation (3). The benefit of an ICD is weighted by the likelihood of receiving therapy from the device for a lethal ventricular tachyarrhythmia against the likelihood of dying from non-arrhythmic causes, prior to receiving device therapy.

Patients with chronic kidney disease (CKD) suffer from high rates of cardiovascular morbidity and mortality with a particular high risk of SCD (4). However, the indication for an ICD in this patient population is less established due to their lack of representation in randomized controlled trials (5–7). Moreover, there is growing evidence that despite the high risk of cardiovascular and sudden cardiac death, patients with advanced CKD are less likely to receive ICD therapies (5, 7, 8). This may be due to their higher risk of dying prematurely from non-arrhythmia related causes (4–6). Furthermore, patients with CKD are more prone to short- and long-term complications associated with ICD implantation that includes bleeding and infection (4, 7).

Due to these concerns the benefit of defibrillator therapy in HFrEF patients with cardiac resynchronization therapy (CRT) and concomitant chronic kidney disease, who are already at high risk of death from other non-arrhythmic causes, remains elusive. In this study, we therefore hypothesized that due to significant competing risks of non-arrhythmic mortality (defined as death occurring prior to an episode of ventricular tachyarrhythmia), patients with CRT and advanced CKD may derive less benefit from an ICD.

## Methods

### Study population

The current study population included patients receiving a cardiac resynchronization therapy with defibrillator (CRT-D) device for primary prevention of SCD who were enrolled in either MADIT-CRT (9) trial or the Ranolazine in High-Risk Patients with Implanted Cardioverter Defibrillator (RAID) trial (10). While not a randomized trial of device implantation, we included the RAID trial because it enabled inclusion of a large number of patients with advanced CKD with a primary prevention ICD receiving contemporary HFrEF therapies. In the RAID trial, treatment with ranolazine was not associated with a significant reduction in the incidence of ventricular arrhythmias or death among patients with HFrEF. Patients with New York Heart Association (NYHA) functional class IV, unknown NYHA classification or unknown ICM status were excluded from this analysis. The studies were conducted from December 2004 through January 2017. The design and results of these trials have been previously reported.

The final study population comprised 1,015 patients with a CRT-D who were divided into two groups based on chronic kidney disease stage as defined by the Kidney Disease: Improving Global Guidelines (KDIGO) categorization (11). The first group included patients with Stage 1 to S3a kidney disease, labeled as (S1-S3a)KD and had an estimated glomerular filtration rate (eGFR) of ≥45 ml/kg/m^2^. The second group included patients with Stage 3b to stage 5 kidney disease, labeled as (S3b-S5)KD and had an eGFR of <45 ml/kg/m^2^.

### Arrhythmia adjudication

All device therapies delivered in each of the trials were blindly adjudicated by at least two experienced electrophysiologists.

### Definitions and endpoints

The primary endpoint of this study was Any VT/VF defined as any device recorded, treated, or monitored sustained VT ≥ 170 beats per minute (bpm) or VF. Secondary endpoints included the following: (1) Appropriate Shock defined as shock therapy for VT ≥ 170 bpm or VF; (2) Fast VT/VF defined as any episode of VT ≥ 200 bpm or VF requiring anti-tachycardia pacing or shock therapy. Death without having experienced any of the above arrhythmic events was treated as a competing event. When discussing the endpoints associated with death without experiencing arrhythmia we refer to those endpoints as non-arrhythmic mortality.

Additional endpoints included: (1) Death from Any Cause (2) Cardiac Death (3) Non-Cardiac Death (4) SCD and (5) Non-SCD.

### Statistical analysis

Continuous variables are expressed as mean ± standard deviation. Categorical data are summarized as frequencies and percentages. We used cumulative incidence function (CIF) curves to estimate and display the probability of patients who developed the event of interest or the competing event as time progressed. Fine and Gray regression modeling, inserting relevant clinical characteristics using a stepwise approach, was employed to identify the optimal model predicting the primary endpoint. A *p* < 0.05 was determined as sufficient to enter the final model. The covariates that were selected were used as adjustment variables in all subsequent models. We additionally adjusted for covariates with clinical significance. The Fine and Gray model of the sub-distribution hazard was employed to evaluate the association of covariates with outcomes that were assessed using the CIF curves. All regression models were stratified by study. We further created extended Fine and Gray models to test the interaction with time and left bundle branch block. The Kaplan–Meier (KM) method was used to calculate the rates of death from any cause, cardiac-death, non-cardiac death, SCD, and non-SCD during 4 years of follow up. Cox-regression models adjusted for the selected covariates in the competing risks models were performed to generate hazard ratios. Note that all hazard ratios that are reported are adjusted for interaction with time due to the violation of the proportional hazards models that were seen in the CIF and KM curves. All hypothesis tests were two sided, with a pre-specified significance level of 0.05. Statistical analysis was performed using SAS version 9.4 (SAS Institute Inc., Cary, North Carolina).

## Results

### Baseline clinical characteristics

**Figure 1:**
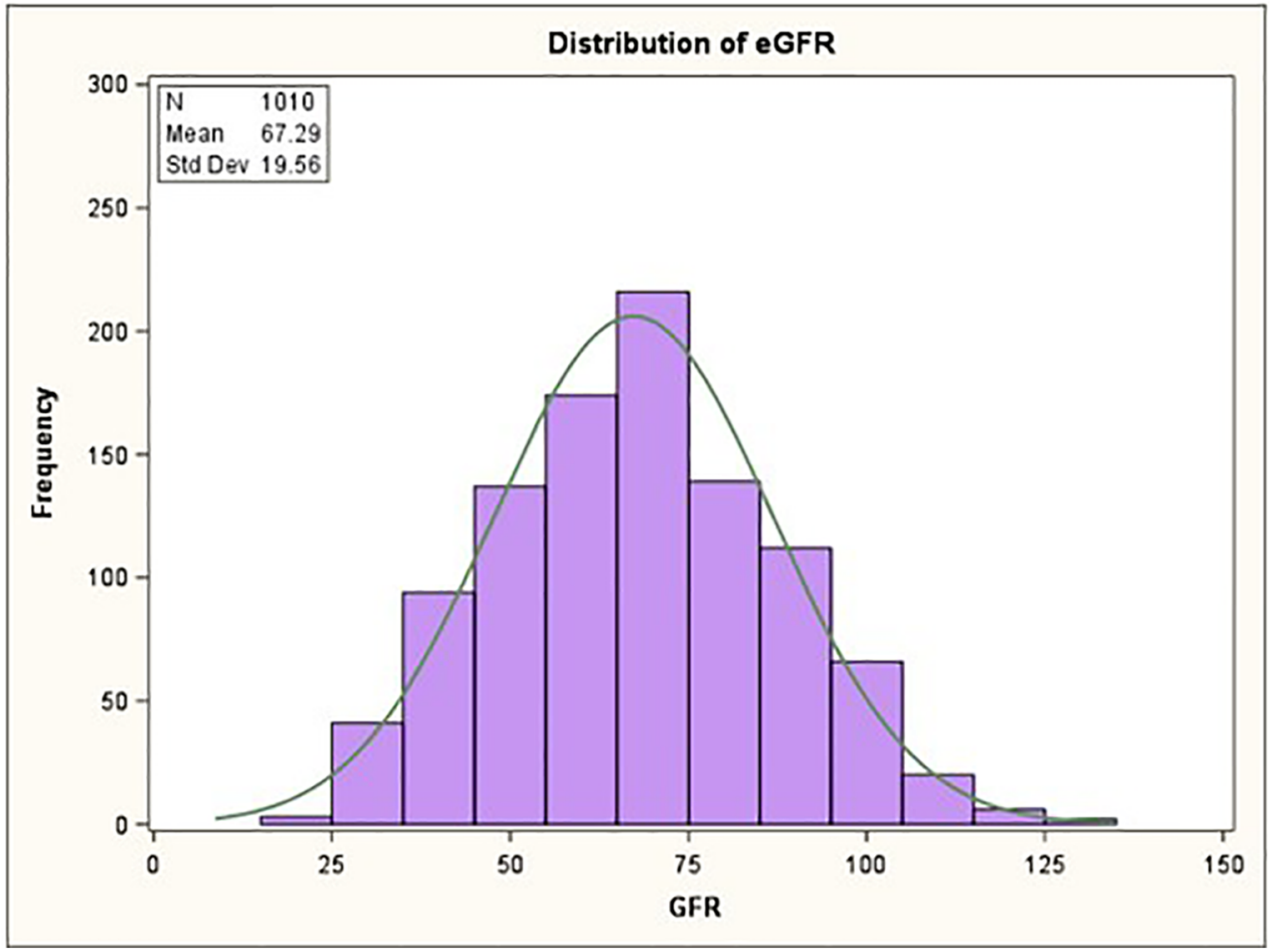
Distribution of estimated glomerular filtration rate (eGFR).

The baseline clinical characteristics by kidney function [(S1-SS3a)KD vs. (S3b-S5)KD] is shown in Table 1. Patients with (S1-SS3a)KD(S1-S3a)KD tended to be younger than those with (S3b-S5)KD(S3b-S5)KD with a mean age of 64 ± 11 vs. 72 ± 8, respectively (*p* < 0.001). Additionally, patients with (S1-SS3a)KD(S1-S3a)KD weighed more at baseline than patients with (S3b-S5)KD(S3b-S5)KD with a mean weight of 86 kg ± 19 vs. 81 kg ± 16, respectively (*p* = 0.017). Patients with (S3b-S5)KD(S3b-S5)KD also tended to have significantly more hospitalizations for heart failure than patients with (S1-SS3a)KD(S1-S3a)KD [23% vs. 16%, respectively (*p* = 0.027)]. Otherwise, there were no significant differences between the two groups in the prevalence of left bundle branch block (LBBB), left ventricular ejection fraction (LVEF), antiarrhythmic drug at baseline, and New York Heart Associated Functional Class. The distribution of GFR in the study population follows a normal pattern with a mean of 67 ml/kg/m^2^ and a standard deviation (SD) of 20 (Figure 1). The median and interquartile range (IQR) for eGFR of patients with (S1-S3a)KD was 71 ml/kg/m^2^and and 60–84, respectively. The median and IQR for eGFR for patients with (S3b-S5)KD was 39 ml/kg/m^2^ and 33–42, respectively (Supplementary Figure S1).

**Table 1:** Baseline clinical characteristics of the study population according to stage of kidney disease.

Clinical characteristic	(S1-S3a)KD	(S3b-S5)KD	*P*-value
	*N* = 864	*N* = 146	
Age at enrollment, mean (±SD)	64 (±11)	72 (±8)	**<** **.** **001**
Female sex	209 (24%)	43 (29%)	0.174
Left bundle branch block	611 (71%)	99 (68%)	0.477
History of non-sustained VT	65 (8%)	10 (7%)	0.741
Weight in kilograms, mean (±SD)	86 (±19)	81 (±16)	**0**.**017**
ACE or ARB	829 (96%)	133 (91%)	**0**.**011**
EF ≤ 25	550 (64%)	87 (60%)	0.346
NYHA ≥ 2	739 (86%)	130 (89%)	0.258
Previous hospitalization	511 (59%)	97 (66%)	0.096
Antiarrhythmic drug at baseline	68 (8%)	18 (12%)	0.074
Beta blocker medication at baseline	812 (94%)	131 (90%)	0.056
History of CHF hospitalization	137 (16%)	34 (23%)	**0**.**027**
Smoking at baseline	102 (12%)	10 (7%)	0.089
Diabetes mellitus	257 (30%)	53 (36%)	0.114
Ejection fraction (%), mean (±SD)	24 (±5)	24 (±5)	0.252
Ischemic cardiomyopathy	461 (53%)	96 (66%)	**0**.**005**
Body mass index, mean (±SD)	29 (± 5)	28 (±5)	0.097
Non-ischemic cardiomyopathy	403 (47%)	50 (34%)	**0**.**005**

Bold indicates statistically significant *P* values <0.05.

### Incidence of arrhythmic endpoints by kidney function

**Figure 2:**
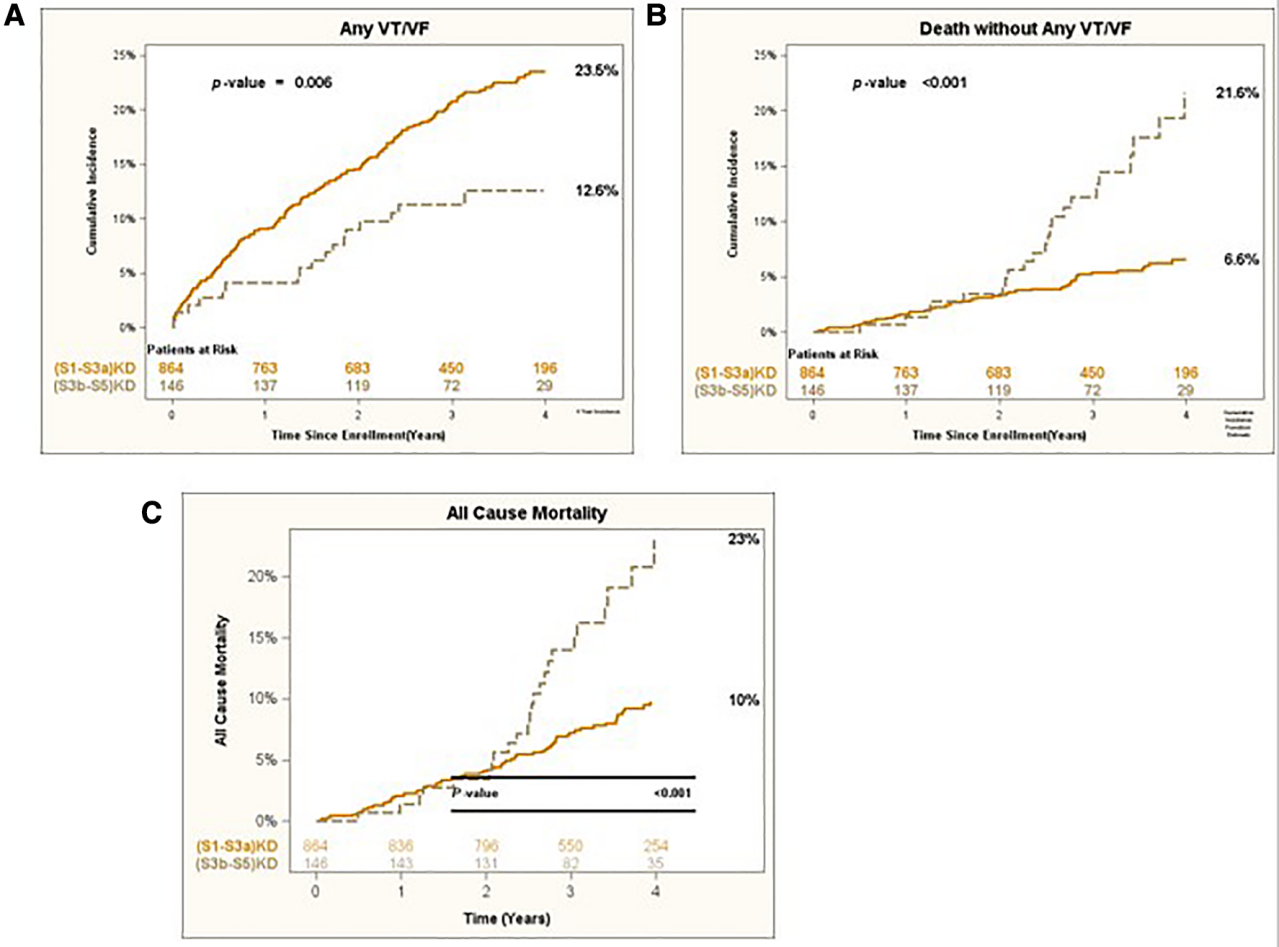
(**A**) Any VT/VF, (**B**) death without any VT/VF, and (**C**) all-cause mortality in early vs. advanced kidney disease after 4 years of follow-up.

After 4 years of follow-up, the cumulative incidence of Any VT/VF was 23.5% in patients with (S1-S3a)KD and 12.6% in those with (S3b-S5)KD with a significant *p*-value of 0.006 for the overall comparison (Figure 2A). In contrast, the incidence of Death without the occurrence of Any VT/VF was 6.6% in patients with (S1-S3a)KD and 21.6% in patients with (S3b-S5)KD with a highly significant *p*-value of *p* < 0.001 (Figure 2B). When evaluating all-cause mortality, the rates of death were 10% and 23% among in patients with (S1-S3a)KD compared to those with (S3b-S5)KD, respectively (*p* < 0.001) (Figure 2C).

**Figure 3:**
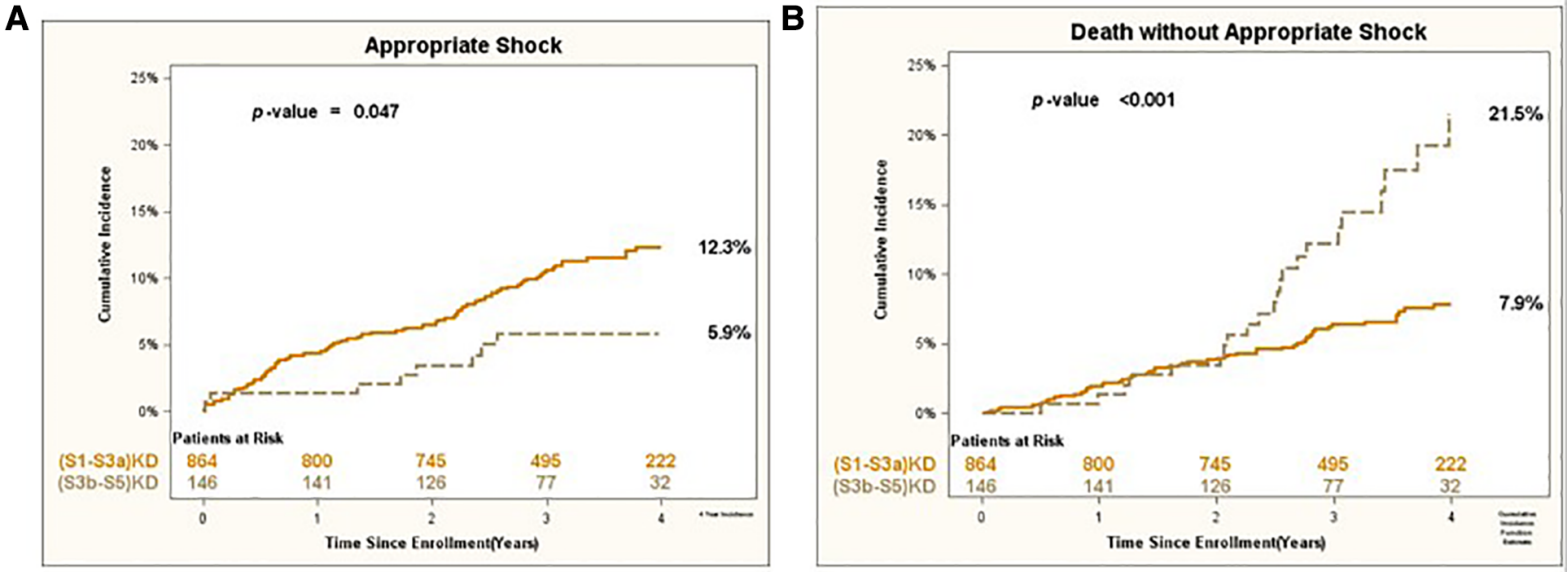
(**A**) Appropiate shock and (**B**) death without appropriate shock in early vs. advanced kidney disease after 4 years of follow-up.

**Figure 4:**
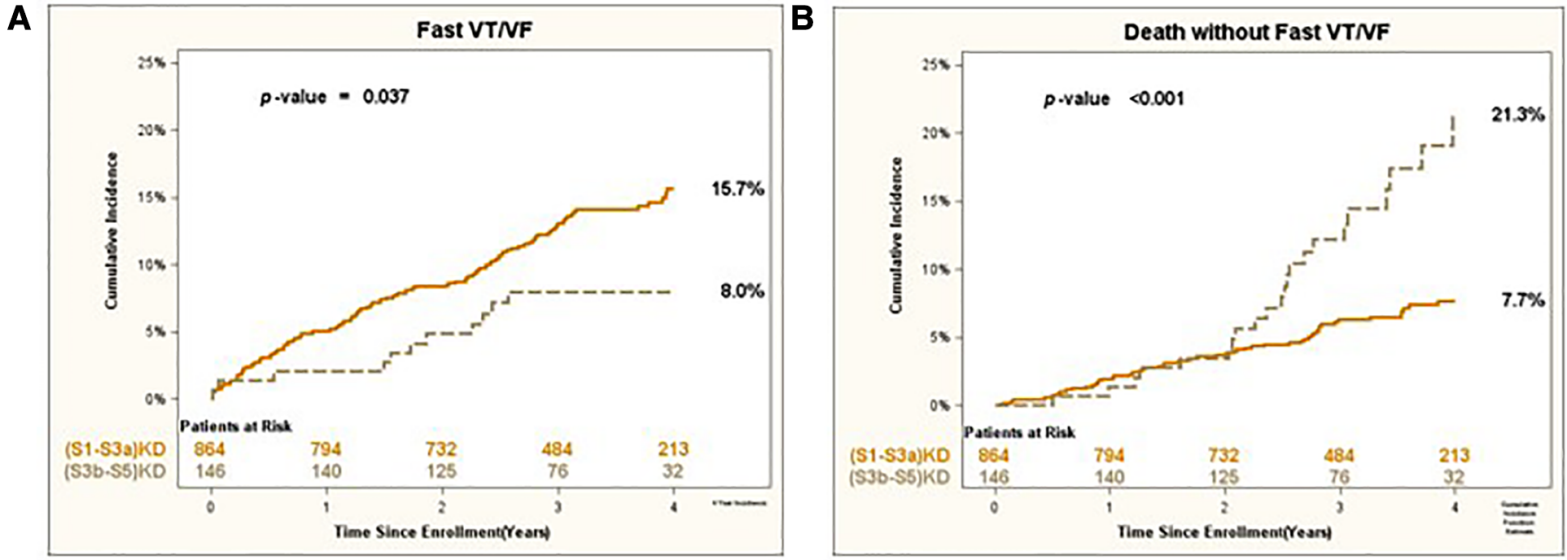
(**A**) Fast VT/VF and (**B**) death without fast VT/VF in early vs. advanced kidney disease after 4 years of follow-up.

Consistent results were obtained for the arrhythmic endpoints of Appropriate Shock [cumulative incidence of 12% vs. 6%; *p* < 0.05 (Figure 3A)] and Fast VT/VF [cumulative incidence of 16% vs. 8%; *p* = 0.037 (Figure 4A)] when comparing patients with (S1-S3a)KD to those with (S3b-5)KD. Notably, a significantly higher cumulative incidence of Death without Appropriate Shock (8% vs. 22%; *p* < 0.001 [Figure 3B) and Death without Fast VT/VF [7% vs. 22%; *p* < 0.001 (Figure 4B)] was observed after 2 years in patients with (S3b-5)KD when compared to those with (S1-S3a)KD.

**Figure 5:**
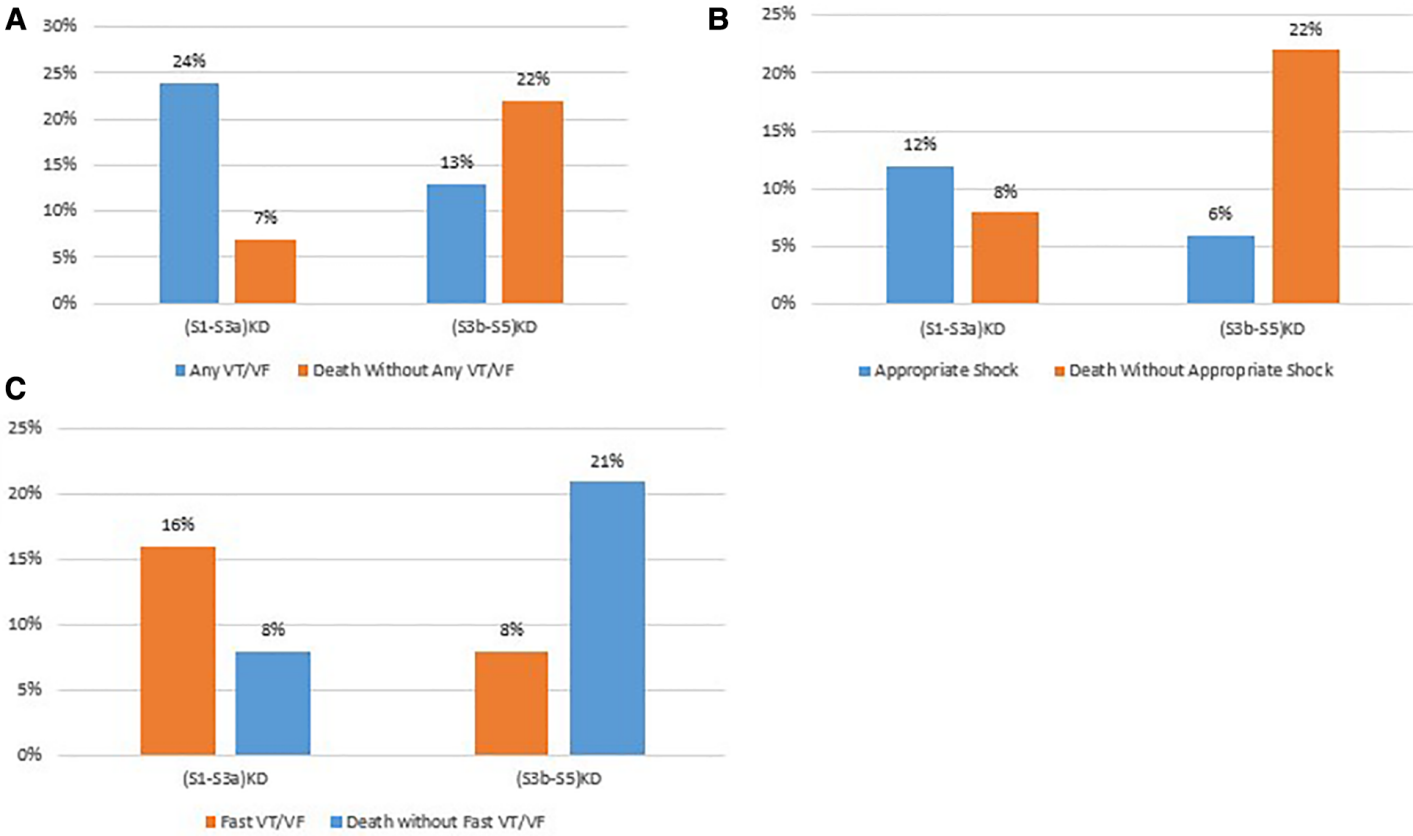
Comparison of arrhythmic vs non-arrhythmic mortality by endpoint. (A) Any VT/VF vs death without any VT/VF. (B) Appropriate stock vs. death without appropriate shock. (C) Fast VT/VF vs death without fast VT/VF.

The rates of arrhythmias and non-arrhythmic mortality at 4 years of follow up by kidney disease group are summarized in Figure 5.

### Regression analysis of first arrhythmic event and non-arrhythmic mortality by kidney function

A Fine and Gray multivariate regression model was developed for the primary and secondary endpoints as described above. These models were further adjusted for sex, diabetes mellitus, ischemic cardiomyopathy, left bundle branch block, self-reported Black race, and age ≥ 65 years. Patients with (S3b-S5)KD had a significant 43% less risk of experiencing Any VT/VF when compared to those with (S1-S3a)KD (HR = 0.56, 95% CI [0.33–0.94] *p* = 0.03 [Table 2A]). Patients with (S3b-S5)KD had a non-significant trend towards a 41% reduction in the incidence of Appropriate Shock [HR = 0.59; *p* = 0.162 (Table 2B)] and a reduction in the risk of Fast VT/VF [HR = 0.67; *p* = 0.214 (Table 2C)].

**Table 2:** Risk of arrhythmic endpoints in advanced compared to early kidney disease after 4 years of follow-up.

Endpoint	Hazard ratio	95% confidence interval	*P*-value
(A) Any VT/VF	0.56	0.33–0.94	**0** **.** **0288**
(B) Appropriate shock	0.59	0.28–1.24	0.1622
(C) Fast VT/VF	0.67	0.35–1.26	0.2140

The reference group for hazard ratios is (S1-S3a) KD.

These models were further adjusted for sex, diabetes mellitus, ischemic cardiomyopathy, left bundle branch block, self-reported black race, and age ≥ 65 years.

Bold indicates statistically significant *P* values <0.05.

When evaluating the competing endpoints of death without arrhythmias, the results were provided with a *p*-value for interaction with time. Prior to two years of follow up there was no significant difference in the incidence of Death without Any VT/VF between patients with (S3b-S5)KD vs. (S1-S3a)KD. In contrast, after two years of follow up there was almost a 5-fold increased risk of Death without Any VT/VF among patients with (S3b-S5)KD when compared to those with (S1-S3a)KD (HR = 4.63, 95% CI [2.46–8.72], *p* for interaction with time = 0.012 [Table 3A]). After 2 years of follow up this risk differential between the two groups was consistent for the endpoints of Death without Appropriate Shock [HR = 3.91; *p* for interaction with time = 0.010 (Table 3B)] and Death without Fast VT/VF [HR = 3.82; *p* value for interaction with time = 0.017 (Table 3C)].

**Table 3:** Non-arrhythmic mortality endpoints in advanced compared to early kidney disease after 4 years of follow-up.

Endpoint	Comparison of KD groups before and after 2 years	Hazard ratio	95% confidence interval	Interaction *P*-value
(A) Death without any VT/VF	Before 2 years	0.98	0.37–2.57	0.012
	After 2 years	4.63	−8.72	
(B) Death without appropriate shock	Before 2 years	0.82	0.32–2.11	0.010
	After 2 years	3.91	−7.21	
(A) Death without fast VT/VF	Before 2 years	0.83	0.32–2.16	0.017
	After 2 years	3.82	2.07–7.04	

The reference group for hazard ratios is (S1-S3a) KD.

These models were further adjusted for sex, diabetes mellitus, ischemic cardiomyopathy, left bundle branch block, self-reported black race, and age ≥ 65 years.

As patients with LBBB tend to respond better to CRT treatment, we also evaluated the interaction between LBBB and the stage of kidney disease. We found that there were no interactions between the stage of kidney disease and the endpoints of Any VT/VF, Appropriate Shock, and Fast VT/VF with *p*-values for interaction of 0.562, 0.881, and 0.788, respectively. Additionally, there was no significant interaction between the stage of kidney disease and cause specific mortality endpoints.

### Mortality rates after 4 years of follow up

**Figure 6:**
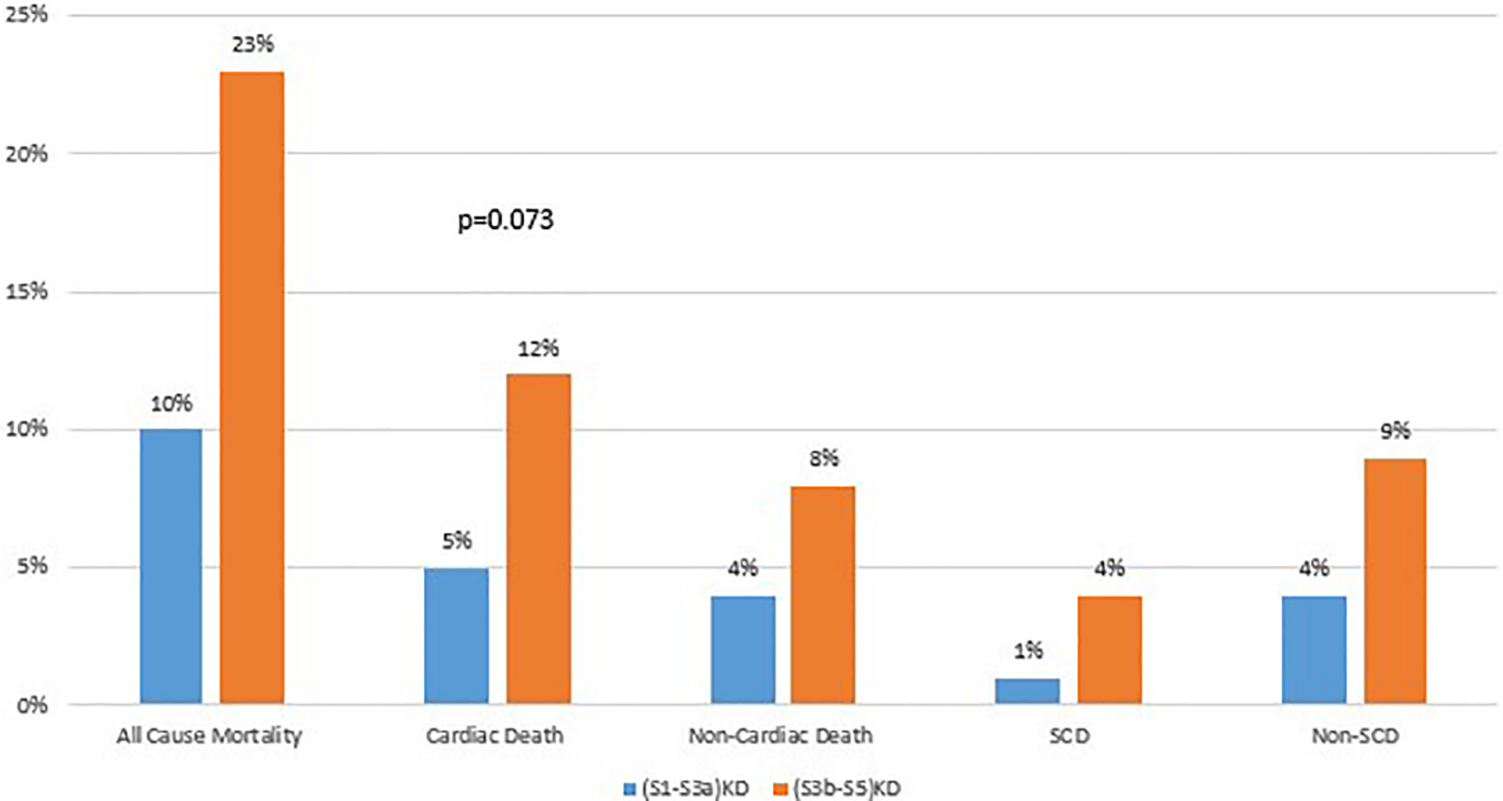
Mortality endpoints after 4 years of follow-up.

The incidence of cardiac death in patients with Stage 3B-5 vs. those with (S1-S3a)KD were 5% vs. 12% over 4 years of follow up (Figure 6), respectively. Consistently, multivariate regression modeling showed that the risk of cardiac death was 3.5-fold higher in patients with (S3b-S5)KD when compared to those with (S1-S3a)KD (HR = 3.46 95% CI [1.56–7.69 *p* = 0.002 (Table 4A)]. A similar trend was seen for the endpoint of Non-Sudden Cardiac Death (9% vs. 4% (Figure 6); HR = 3.48 95% CI [1.41–8.61] *p* = 0.007[Table 4B). However, there was only a trend toward an increased risk for the endpoint of SCD that did not meet statistical significance when comparing the (S3b-S5)KD group with the (S1-S3a)KD group (4% vs. 1% (Figure 6); HR = 3.66 95% CI [0.67–19.91] *p* = 0.133 [Table 4C]). Finally, the risk of Non-Cardiac Death was also higher in the (S3b-S5)KD group when compared to the (S1-S3a)KD group without meeting statistical significance (8% vs. 4% (Figure 6); HR = 2.10 95% CI [0.84–5.25] 0.111 [Table 4D]).

**Table 4:** Cause specific mortality endpoints after 4 years of follow-up.

Endpoint	Group comparison	Hazard ratio	95% confidence interval	*P*-value for interaction with time
All-cause mortality	Before 2 years	0.82	0.32–2.11	0.034
	After 2 years	3.19	1.83–5.57	
Cardiac death	Before 2 years	0.38	0.05–2.83	0.025
	After 2 years	3.46	1.56–7.69	
Non cardiac death	Before 2 years	1.10	0.31–3.91	0.383
	After 2 years	2.10	0.84–5.25	
SCD	Before 2 years	0.00	0.00–0.00	0.141
	After 2 years	3.66	0.67–19.91	
Non SCD	Before 2 years	0.42	0.06–3.14	0.055
	After 2 years	3.48	1.41–8.61	

The reference group for hazard ratios is (S1-S3a) KD.

These models were further adjusted for sex, diabetes mellitus, ischemic cardiomyopathy, left bundle branch block, self-reported black race, and age ≥ 65 years.

### Cause specific mortality

**Figure 7:**
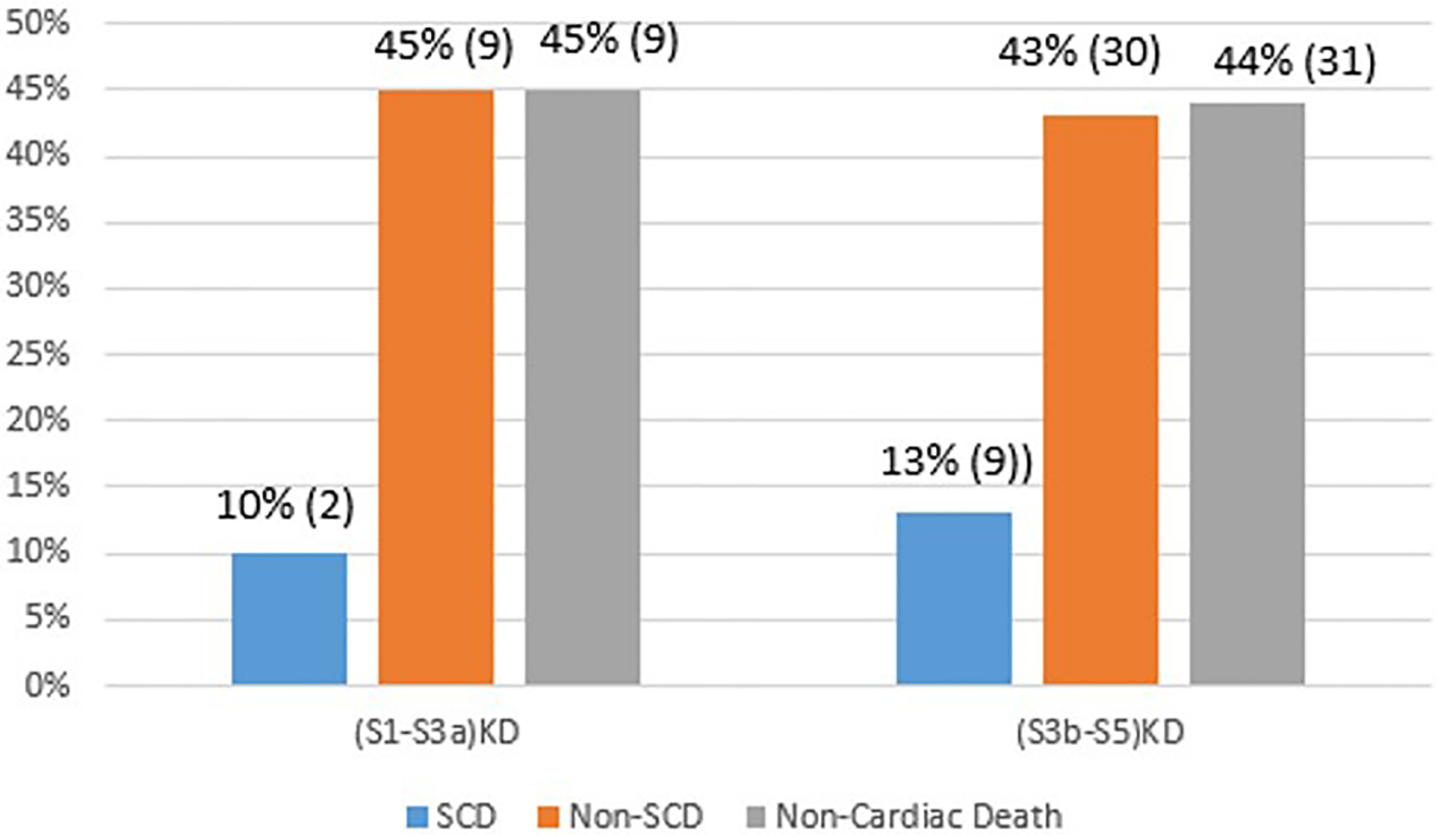
Cause specific mortality after 4 years of follow-up.

Cause specific proportions of deaths in each KD group are illustrated in Figure 7. In the (S1-S3a)KD group 10%, 45%, and 45% of deaths constituted SCD, Non-SCD, and Non-Cardiac Death, respectively. In the (S3b-S5)KD group 13%, 43%, and 44% of deaths constituted SCD, Non-SCD, and Non-Cardiac Death, respectively.

## Discussion

In this study incorporating a large number of patients from 2 major randomized clinical trials, we found that CRT device recipients who are implanted with a primary prevention ICD and have (S3b-S5)KD tend to experience a significantly lower risk of Any VT/VF when compared to patients with (S1-S3a)KD while sustaining a much higher risk of non-arrhythmic mortality that becomes apparent after two years of follow up.

CKD is often a common comorbidity in patients with heart failure and is associated with increased incidence of both cardiovascular morbidity and mortality. Treatment with CRT has been shown through randomized clinical trials and registry data to have a beneficial effect on the disease course of patients with HFrEF and CKD who meet the appropriate device indications (9). However, data regarding the benefit of an ICD in this patient populations remains elusive partly because of lack of representation of such patients in RCTs involving the ICD. Additionally, patients with CKD remain at increased risk of competing non-cardiac risks of death. While CRT is helpful in this patient population, the addition of an ICD is not trivial and could be associated with a risk of inappropriate shocks, shorter battery life, higher procedural complications and costs.

While an ICD for secondary prevention of SCD has been suggested to be associated with improved survival in patients with ESRD (12), its benefit in patients with a primary prevention indication remains elusive (13, 14). Our results provide a unique perspective as to why a primary prevention ICD may not be beneficial in this patient population. Even though there is evidence in the literature that patients with CKD are at particularly high risk of sudden cardiac death, we found that there was no difference between the risk of life-threatening ventricular arrhythmias in patients with (S1-S3a)KD vs. those with (S3b-S5)KD. This suggests that sudden death in this patient population may have a greater proportion of non-arrhythmic sudden death including pulseless electrical activity and asystole. We additionally showed that the risk of sudden cardiac death is 4% vs. 1% in patients with (S1-S3a)KD vs. those with (S3b-S5)KD, respectively. When evaluating non-sudden cardiac death, the difference is much greater with a rate of 9% in the (S3b-S5)KD group and 4% in the (S1-S3a)KD group.

It is reported in the literature that more than two-thirds of mortality in CKD stages are a result of sudden death, and sudden death is mainly caused by ventricular arrhythmias (15). However, this disproportionate increase in sudden cardiac death may be driven by end-stage renal disease (ESRD) patients on hemodialysis (15). In fact, sudden death was the major cause of death in patients with ESRD in one study reaching 50% while the proportion of SCD was 10.1% and 10.3% in patients with GFR < 60 ml/min and GFR ≥ 60 ml/min, respectively (15). The cause-specific mortality rates in that study are comparable to the rates observed in our study among patients implanted with a CRT-D. These findings suggest that in patients with (S3b-S5)KD implanted with a CRT who have not yet reached the stage of hemodialysis, optimizing medical therapy with a focus on prevention of non-sudden causes of death and non-arrhythmic mortality could have major benefit on the course of their disease.

The pathophysiology of increased propensity for ventricular arrhythmias in patients with CKD has been mainly described in patients receiving hemodialysis. This may be secondary to volume and sudden electrolyte shifts during dialysis (16). More recently, a study showed that indoxyl sulfate, a uremic toxin, has an arrhythmogenic effect in cardiomyocytes *in vitro* (16)*.* An association was found between its levels and QT interval prolongation in CKD patients not on dialysis. Another potential arrhythmogenic mechanism in patients with CKD is the elevated level of parathyroid hormone (PTH) which has been linked to the occurrence of SCD. The interplay of anemia, inflammation, and mineral and bone disorders is likely associated with increased arrhythmic risk in those patients with advanced CKD (16).

Conceptually, those patients who die prior to experiencing a ventricular arrhythmic event or therapy from the ICD are less likely to benefit from it. Our results consistently illustrate through CIF curves and multivariate Fine and Gray regression models that although the risk of non-arrhythmic mortality is similar between patients with (S1-S3a)KD and (S3b-S5)KD for the first two years, after two years of follow up the latter group experiences a significantly higher risk of dying prior to receiving an arrhythmia or device therapy. These findings are important to clinical practice as once a diagnosis of heart failure is established among patients with late stages of CKD, there is a time period to fully optimize medical therapy and treat comorbidities before there is a disproportionate increase in the risk of non-arrhythmic mortality.

Current guidelines recommend implantation of an ICD in patients with a life expectancy of at least a year (17). Therefore, it may seem reasonable to utilize it for the prevention of SCD in patients with advanced stages of CKD as the rate non-arrhythmic mortality is similar to that of patients with earlier stages of CKD. However, our results show that the non-arrhythmic mortality risk dramatically increases after two years of follow up in patients with advanced CKD which may limit long term benefit from the ICD.

The Defibrillator Implantation *n* Patients with Nonischemic Systolic Heart Failure (DANISH) trial (18) suggested that a select group of patients with HFrEF may benefit from a CRT alone (CRT-P) rather than a CRT-D. The results of that trial suggested that younger patients may have a survival benefit in association with the ICD (18). This concept is similar to patients with (S3b-S5)KD who may benefit less from the ICD due to not living long enough to accrue clinical benefit from the device. Currently there is insufficient evidence to argue against implantation of a defibrillator in patients with (S3b-S5)KD who receive CRT treatment. To date the only trial that randomized patients to a CRT-D arm vs. a CRT-P arm was the Cardiac-Resynchronization Therapy with or without an Implantable Defibrillator in Advanced Chronic Heart Failure (COMPANION) trial, and it was only powered to compare each of these arms to medical therapy alone but not to each other (19). A post-hoc analysis of this trial showed no significant difference in outcomes between CRT-D and CRT-P recipients with NYHA Class IV symptoms. Future trials are needed to better assess the impact of CRT-P vs. CRT-D in patients with CKD. Based on the results of our study we hypothesize that due to their comparatively high mortality rate and low rate of ventricular arrhythmias, patients with advanced kidney disease may not receive significant survival benefit from a defibrillator. Additionally, with the advent conduction system pacing and left bundle area pacing, the difference between CRT-P and CRT-D may be more significant in that left bundle area pacing has the unique advantage of restoring cardiac resynchronization without the need for placement of an ICD lead nor a coronary sinus lead (20).

There may be several mechanisms that lead patients with advanced CKD to experience less arrhythmias than those with early CKD despite their higher risk of sudden cardiac death that is described in the literature (15). First, it is possible that among patients with advanced CKD, ventricular arrhythmias are more fatal since they are less responsive to ICD therapies such as anti-tachycardial pacing or appropriate shocks. This is only a hypothesis as we did not capture data on device interrogation at the time of death. Another possible mechanism for this phenomenon is that because patients with advanced CKD also have a greater competing risk of dying from other causes associated with their more advanced medical illness (13). This could potentially lead to such patients not living long enough to experience an arrhythmia and thus benefit from the ICD. In contrast in patients with less advanced kidney disease, the overall risk of death is lower and are therefore more susceptible to the occurrence of ventricular arrhythmias during long-term follow-up after primary device implantation.

Our study has several limitations that require recognition. First, the analysis was performed in a *post hoc* fashion, combing data of patients with HFrEF receiving a CRT-D who were enrolled in 2 previously conduced trials and therefore the findings of our study should be considered hypothesis generating. Second, we did not have follow-up measurements of renal function and therefore a time dependent analysis of renal function on arrhythmia and non-arrhythmic mortality outcomes could not be carried out. Third, contemporary treatment of HFrEF patients has evolved and now includes novel therapeutic agents such as sodium glucose contransporter-2 inhibitors (SGLT2-I) (21) and angiotensin receptor neprilysin inhibitors (22) and as a result the impact of these therapies on arrhythmic outcomes in patients with renal dysfunction could not be assessed. Recent data suggests that SGLT2-I (23, 24) dapagliflozin has renal protective effects in addition to its hemodynamic effects. Finally, after a 2 year period of follow up cardiac death and SCD became higher. Cardiac death is the combination of SCD and non-SCD. The cause SCD is presumably due to an arrhythmia and non-SCD is presumably due to heart failure. Unfortunately, we do not have the detail of death beyond the specification of sudden vs. non-sudden cardiac death.

### Conclusion and clinical implications

In conclusion, the results of this study suggest that due to their comparatively high non-arrhythmic mortality rate and lower rate of ventricular arrhythmias, CRT recipients with advanced CKD do not appear to attain the same benefit from a primary prevention ICD as those patients without advanced CKD. Given the associated complications and added costs associated with an ICD, future randomized trials should determine whether a CRT-P is non-inferior to a CRT-D in patients with advanced CKD.

## Data Availability

The data analyzed in this study is subject to the following licenses/restrictions: The datasets are the property of the University of Rochester Medical Center. Requests to access these datasets should be directed to ilan.goldenberg@heart.rochester.edu.
