# Supplementary figures and images for: Primary prevention implantable cardioverter defibrillator in cardiac resynchronization therapy recipients with advanced chronic kidney disease
Source: Front Cardiovasc Med. 2023 Aug 23;10:1237118. doi: 10.3389/fcvm.2023.1237118 (PMC10482044; doi:10.3389/fcvm.2023.1237118)

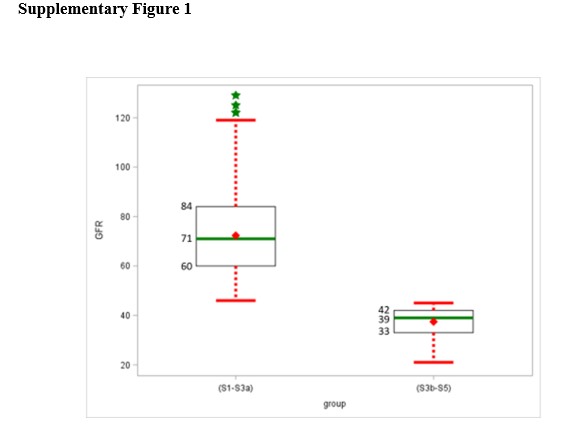

Supplement: Supplementary file 1 [file Image1.jpeg]
